# EzrA: a spectrin-like scaffold in the bacterial cell division machinery

**DOI:** 10.15698/mic2015.02.187

**Published:** 2015-01-15

**Authors:** Robert Cleverley, Richard Lewis

**Affiliations:** 1 Institute for Cell and Molecular Biosciences, University of Newcastle, Newcastle-upon-Tyne, NE2 4HH, UK.

**Keywords:** cytoskeleton, cell division, spectrin, EzrA, crystallography

## Abstract

Much progress has been made in identifying the components of the divisome, the assembly of proteins that undertakes the vital process of cell division in bacteria. However, how the highly interdependent processes on either side of the membrane are coordinated during division is a major unresolved question. How is the degradation and synthesis of the cell wall on the outside of the cell coordinated with cytokinesis and membrane fission, which are driven from the inside of the cell by the tubulin homologue FtsZ? A possible key mediator of such coordination is the membrane protein EzrA, as it interacts both with FtsZ and the penicillin binding proteins (PBPs) that synthesize peptidoglycan. Cleverley *et al.* [Nature Communications (2014) 5, 5421] have recently solved the crystal structure of the cytoplasmic domain of *B. subtilis* EzrA, which points to an important scaffolding role for EzrA in the divisome. The structure resembles the eukaryotic, cytoskeletal spectrin proteins, which link actin filaments in the cytoskeleton and also connect the actin cytoskeleton to membrane-bound integrin proteins.

EzrA contains a single transmembrane helix at its N-terminus followed by the 540 amino acid cytoplasmic domain. The structure of the *B. subtilis *EzrA cytoplasmic domain reveals an extended rod with three alpha helices packed together along its length. The rod comprises five repeats of a ~100 amino acid triple helical bundle, connected in a head-to-tail fashion, and then terminates with a four helical bundle (**Figure 1**). Such a head-to-tail interlinkage of repeating triple helical bundles is a defining characteristic of spectrin proteins and indeed four of the five *B. subtilis *EzrA repeat units can be structurally superimposed on the equivalent units in representative structures of spectrins.

**Figure 1:**
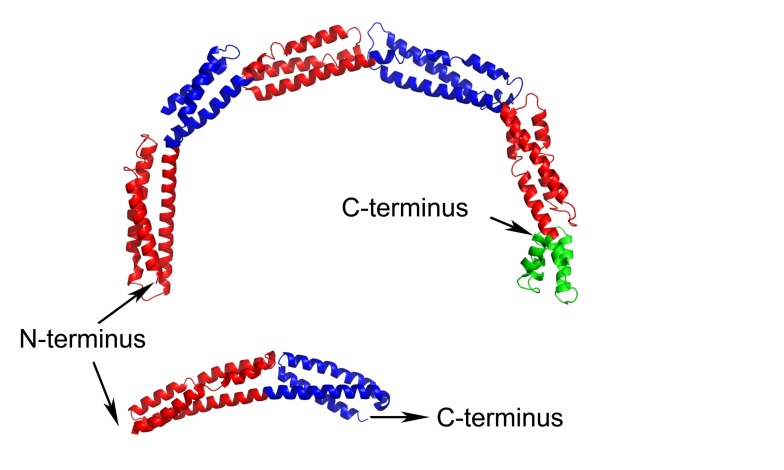
FIGURE 1: Structures of *B. subtilis* (top) and *S. aureus* (bottom) EzrA cytoplasmic domains. The five ~100 amino acid triple helical bundle repeat units in *B. subtilis* EzrA are coloured alternately red and blue; the terminal 4 helical bundle is coloured green. The *S. aureus* EzrA structure is an N-terminal fragment of the cytoplasmic domain, encompassing just two of the five triple helical bundle units.

While the coiled-coil-like fold of EzrA had been predicted from its primary sequence, the resemblance to spectrins was completely unanticipated. To date, a convincing bacterial homologue of the spectrins has not been identified, either at the structural or sequence level. This lack of sequence homology is explained by a subtle difference between the structures of EzrA and spectrins. While the EzrA and spectrin repeat units have the same topological up-down-up arrangement of three helices, the connectivity between the helices within the unit differs. Spectrins have a left-handed connectivity - when viewed end-on along the principal axis of each repeat, the first and third helices are related by an anti-clockwise rotation. By contrast, the EzrA repeat unit has the opposite, right-handed, connectivity. As a result the packing interactions amongst the helices in each repeat differ in the two protein families.

A remarkable, unprecedented feature of the *B. subtilis *EzrA structure is that the rod has a continuous curvature so that it has an overall semi-circular shape, with a diameter of 120 Å. This has fascinating implications as regards to the positioning of the cytoplasmic domain in the divisome. In the simplest model, supported by sequence analysis, the N-terminal transmembrane helix represents a straight extension of the first helix in the cytoplasmic domain. With a standard perpendicular orientation of this TM helix in a straight lipid bilayer the EzrA semi-circle will form a complete arch over the surface of the membrane (**Figure 2**), with the C-terminal helical bundle close to the surface of the membrane. This would enable EzrA to form long-distance connections between adjacent membrane-associated proteins. Intriguingly, the arc could also act as a hook trapping divisome components in the gap between the membrane surface and the inside of the arc. Molecular modelling reveals there is sufficient space to accommodate an FtsZ filament in this gap and even to accommodate simultaneously both FtsZ and its membrane anchor FtsA (**Figure 2**).

**Figure 2:**
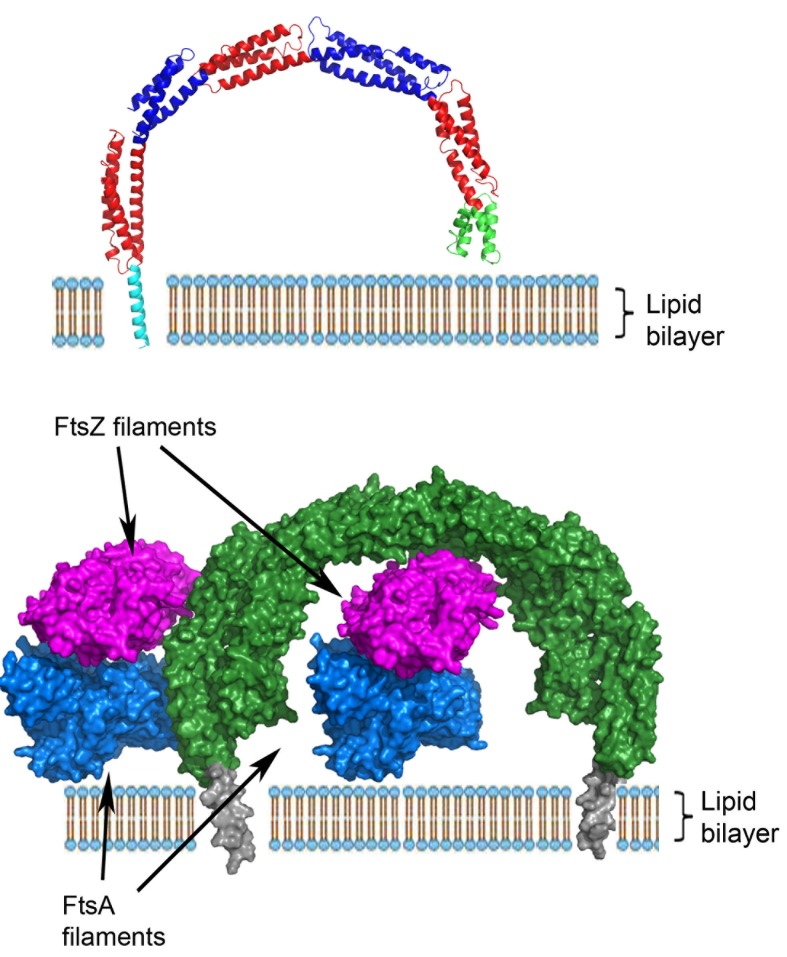
FIGURE 2: **Top - ** Model of the integration of *B. subtilis *EzrA into the lipid bilayer. The modelled N-terminal helix is coloured cyan, the remainder of the cytoplasmic domain is coloured as in Figure 1. **Bottom - ** Model for the interaction of EzrA (green, with the modelled TM helix grey) with FtsZ protofilaments (magenta) and the FtsZ membrane anchor, FtsA (blue) within the divisome; the FtsZ and FtsA filaments are viewed along the long axis of the respective filaments. EzrA is represented in an anti-parallel dimer that is observed in the crystal lattice and is supported by *in vivo* two hybrid studies. There is sufficient space to accommodate both FtsA and FtsZ in the gap between the membrane and the inside of the arch-shaped EzrA molecule.

The notion of EzrA enclosing individual FtsZ filaments can be reconciled with evidence from studies both *in vivo* and *in vitro*, which are consistent with EzrA acting as a negative regulator of FtsZ oligomerization. Both FtsZ and tubulin polymerize first in a longitudinal manner, to form elongated protofilaments. Subsequent lateral interactions between individual protofilaments form higher order filamentous assemblies. It is self-evident that the lateral interactions between adjacent FtsZ protofilaments will be disrupted when they are enclosed by an EzrA molecule.

In summary, the recent structure of EzrA has extended the similarities between the eukaryotic and prokaryotic cytoskeletons to the spectrin family. The structure has important implications with regard to the arrangement of the components of the divisome and, given that EzrA is essential in several pathogens, represents a starting point for the development of novel antibiotics.

